# Developmental Aspects of Schizotypy and Suspiciousness: a Review

**DOI:** 10.1007/s40473-018-0144-y

**Published:** 2018-02-09

**Authors:** Keri K. Wong, Adrian Raine

**Affiliations:** 1 Department of Psychology, University of Cambridge, Cambridge, UK; 2 Cambridge, Cambridgeshire UK; 3 Departments of Criminology, Psychiatry, and Psychology, University of Pennsylvania, Philadelphia, PA USA

**Keywords:** Schizotypal personality disorder, Schizophrenia, Paranoia, Suspiciousness, Dimensional, Developmental

## Abstract

**Purpose of the Review:**

This review identifies the early developmental processes that contribute to schizotypy and suspiciousness in adolescence and adulthood. It includes the most recent literature on these phenomena in childhood.

**Recent Findings:**

The early developmental processes that affect schizotypy and paranoia in later life are complex. In contrast to existing studies of psychiatric patients and clinical/nonclinical adult populations, the study of schizotypy and suspiciousness in young children and adolescents is possible due to new child-appropriate dimensional assessments. New assessments and the advancement of technology (e.g., virtual reality in mental health) as well as statistical modeling (e.g., mediation and latent-class analyses) in large data have helped identified the developmental aspects (e.g., psychosocial, neurocognitive and brain factors, nutrition, and childhood correlates) that predict schizotypy and suspiciousness in later life.

**Summary:**

Prospective longitudinal designs in community youths can enhance our understanding of the etiology of schizophrenia-spectrum disorders and, in the future, the development of preventive interventions by extending adult theories and interventions to younger populations.

## Introduction

Schizophrenia is a disabling, multidimensional disorder affecting roughly 1% of the general population. Studies of patients with persecutory delusions and schizotypal personality disorder (SPD) have helped advance our understanding of schizophrenia over the years, but its causes are still complex and its treatments are limited. Prospective longitudinal studies have recognized that schizotypy and suspiciousness reflect liability for later schizophrenia; hence, the continued research and clinical interest in these phenomena and its attenuated forms may shed light on the causes of schizophrenia. The presence of schizotypy and suspiciousness at both the clinical and community levels are profoundly distressing and associated with negative psychosocial functioning; hence, studying the developmental aspects of schizotypy and suspiciousness are again of significant clinical and research importance.

Like schizophrenia, schizotypal personality disorder is a multidimensional disorder commonly reported to have a three-factor structure: *cognitive-perceptual deficits* (ideas of reference, odd beliefs, perceptual disturbances, and paranoia/suspiciousness), *interpersonal deficits* (lack of close friends, social anxiety, paranoia/suspiciousness), and *disorganization* (odd speech/thought, blunted affect, odd behavior). Schizotypy research informs our understanding of the etiology of schizophrenia in several ways: schizotypy allows researchers to study the prodrome and at-risk states of schizophrenia and related disorders taking a developmental approach. This approach allows the identification of developmental causes along the schizotypy continuum of severity. Schizotypy’s multidimensional framework, which includes paranoia/suspiciousness, enhances our understanding of the heterogeneous etiologies of schizophrenia-spectrum disorders, by allowing differential relationships to be examined across the dimensions. For example, recent findings have recognized that cognitive-perceptual and disorganized features of schizotypy provide high sensitivity and moderate discriminate validity in the SPD diagnosis compared to other personality disorders in the DSM-IV [1].

Paranoia, or excess suspiciousness, is the unfounded belief that others purposely intend to cause harm [2•]. It is a subcomponent of schizotypy and the most commonly reported subtype of delusion in schizophrenic patients. While these subtypes are often interrelated, factor analytic studies have demonstrated that paranoia is a separate type of psychotic experience and therefore merits study in its own right [3]. While studies of suspiciousness have focused primarily on adult patient samples, studies in the last two decades have replicated findings from patient studies in young adults in the general population [4•]. More recent studies have even been extended to young children and adolescent populations in the general population, recognizing that attenuated symptoms of suspiciousness in the form of social mistrust exist early in development and are heritable and also associated with childhood psychopathology ([5••]; Zhou, (Wong KK, Raine A, & Venables P (under review). Being left home alone at age three years is associated with increased schizotypy and antisocial behavior at ages 17 and 23 years.)). A short-term longitudinal study replicated this finding in help-seeking adolescents, identifying that paranoia persistence is predicted by a host of cognitive, affective, and social factors. Thus, studying delusions developmentally in the general population may help understand the clinical condition and develop preventive interventions.

Both schizotypy and suspiciousness exist on a dimension; that is, schizotypy lies on a continuum of severity with schizophrenia and persecutory delusions lies on a continuum with paranoia [5••, 6•, 7•, 8]. Clinical symptoms of schizophrenia extend to milder symptoms of schizotypy and suspiciousness in the general population [2•, 9]. In schizotypy, studies have continuously demonstrated that subclinical psychosis closely resembles symptoms that have been identified in schizophrenia, indicating a continuum between the subclinical and clinical phenotypes [10, 11]. Similarly, in the paranoia literature, a review of 14 studies found that 10 to 15% of non-clinical young adults frequently report paranoid thoughts, of which 3% report clinical levels of paranoid thoughts that go undiagnosed [4•]. A recent meta-analysis of 35 unique participant cohorts has documented a strong support for a psychosis continuum in the population, with a median prevalence rate of approximately 5% and an incident rate of 3% [11]. The authors suggest that 75–90% of developmental psychotic experiences are fleeting and disappear over time; however, there is evidence that some of these developmental psychotic experiences may become more persistent and disabling given an individual’s level of exposure to subsequent environmental risks throughout life. Thus, understanding these developmental risks is of utmost importance in identifying those in need.

Overall, the study of schizotypy and paranoia is well established with a number of independent reviews on schizotypy and suspiciousness [1, 2•, 6•, 9, 12, 13]. However, a few review the developmental aspects of the two conditions simultaneously. The current review fills this gap by presenting the developmental aspects that shape later schizotypy and suspiciousness in adulthood and the initial evidence of these phenomena in children and adolescents. We argue that a developmental perspective can extend our understanding of the etiology of schizophrenia and improve the identification of windows of opportunity for preventative or therapeutic interventions. This review includes both published and works under review on childhood schizotypy and suspiciousness that examine the assessments, developmental causal factors, treatments, and interventions before concluding with an overview of the future directions.

## Assessments

### Clinical Interviews, Questionnaires, and Virtual Reality

Schizotypal personality disorder and paranoia are commonly assessed by both categorical and dimensional approaches. Taking into account an individual’s personal and medical history, a clinician or mental health profession will administer the SCAN 2.1 (Schedules for Clinical Assessment in Neuropsychiatry by the WHO) or the SCID-5 (Structured Clinical Interview for the DSM-5). Both are semi-structured interviews to systematically determine the extent to which an individual qualifies for a mental disorder. The SCID-5 provides a current and lifetime assessment for schizophrenia and psychotic disorders, often administered in conjunction with self-report questionnaires.

Complementing the categorical approach, self-report questionnaires measure dimensional aspects of schizotypy. The Schizotypal Personality Questionnaire (SPQ; [14]) is one of the most widely used measures of schizotypy. It is modeled on the *DSM-III-R* clinical diagnosis of schizotypy, capturing a well-replicated stable 3-factor structure using 74 yes/no items [15]. The SPQ has high internal consistency (*α* = 0.91), test-retest reliability (*r* = .82), convergent validity (*r* = .59 to .81), discriminant validity, criterion validity with the SCID-II (*r* = .63 and .68) [16]. A shorter 22-item version is also available (SPQ-B; [17]) and recently confirmed to be psychometrically robust and valid across 14 international samples of 16 to 68 year olds (*n* = 10,711, omega coefficient = 0.86 to 0.92) [18••]. As both the SPQ and SPQ-B are intended for adults, an equivalent reliable 22-item version SPQ-C with minor modifications is available for children (*α* = 0.91; [19•]). The three-factor structure of schizotypy is found to be stable across time and instrument for the SPQ [15] and SPQ-B [20], international populations of psychiatric patients [21–24], community adults [25–27], and adolescents [19•, 28] and is invariant across gender, religion, ethnicity, and social backgrounds [26, 29]. The SPQ and its subsequent brief versions are psychometrically robust, where a higher score often reflects greater neurobiological and genetic vulnerabilities [30, 31], thus offering researchers a tool to assess the etiology of schizotypy across development.

Commonly used adult measures of paranoia range from 18- to 40-item Likert-scale self-reports and interview schedules assessing the frequency, persistence, levels of conviction and distress, and the severity of the persecutory delusions, where a higher score reflects greater distress on the dimension. A notable feature of these adult instruments is reference to time-span (e.g., “over the past year…,” “in the last week…”), which is often difficult for children to comprehend. Newer childhood measures of suspiciousness have dropped such references to time span. In one of the first child-appropriate dimensional measure of childhood suspiciousness—the Social Mistrust Scale (SMS: [5••])—the suspicion is referenced with school and home environments to assist in children’s recall and the questions kept brief. Validity studies of the SMS are growing in number and have thus far demonstrated good psychometric properties across ages (8 to 18 years alpha = 0.65 to 0.83), cultures (China, Hong Kong, and the UK), and instruments (i.e., convergence with the Positive and Negative Syndrome Scale, *r* = .29 to .32).

Complementing both interviews and questionnaires is virtual reality (VR) technology, which has received much research and clinical attention in psychiatry as it is seen as an effective experimental tool for symptom verification and treating mental health conditions in adult patients [32•]. In particular, VR has been applied as an assessment and treatment tool for patients with persecutory delusions. To date, there have been 44 VR studies on schizophrenia, of which 15 concern the assessment of paranoia, while six use VR as a treatment [33]. In the latest intervention study, Freeman et al.’ [34••] found large effects (*d* = 1.3) for VR cognitive therapy compared to VR exposure therapy in reducing delusions and distress in real-world situations. However, the sample was small (*n* = 30 patients) and researchers were not blind to participant’s treatment assignment.

While virtual reality technology is used alongside traditional measures of paranoia (i.e., questionnaires and clinical interviews) to triangulate findings, VR by itself has several advantages. One prevailing challenge for paranoia research, which VR is able to overcome, is whether or not an individual’s delusion is genuine or unfounded. VR has clear advantages in providing live moment-to-moment assessments of paranoia by engaging the individual with a socially controlled computer-rendered environment with computer characters (e.g., London tube ride). By asking what the individual perceives of the avatars in the simulation, the researcher can gauge the extent to which a response or reaction to a benign avatar is hostile or neutral. A further advantage of the controlled VR environment lies in intervention. VR studies on adult patients with paranoia support the use of VR cognitive therapy in treating persecutory delusions. Through VR, researchers are able to repeatedly test and identify the environmental factors that trigger suspicions, in order to gradually modify the environment to help an individual change and better manage their hostile interpretations over time [34••]. Whether or not virtual therapy is effective in symptom identification and treatment in community population and youths is yet to be investigated.

In summary, the technological advancement in VR and the development of new child-appropriate assessments add to the field by refining existing assessments through the triangulation of different sources of data. Though it remains to be seen whether virtual assessments and therapies may be helpful for assessing attenuated symptoms in younger populations, these advancements open up new developmental research possibilities for researchers to assess large groups of young people in normative samples. These efforts together help identify windows of opportunity for preventative and therapeutic interventions early in child development.

## Causes

The causes of schizotypal personality disorder and suspiciousness are complex. Empirical evidence suggests that there is no single factor responsible but that a multitude of interacting biological, social, and psychological factors are important considerations. We discuss these in turn below.

### Psychosocial Influences and Environmental Adversity

According to evidence from cross-sectional and prospective studies using both self-report and official records, persons high in schizotypy and individuals with psychotic-like symptoms are more likely to report a history of child abuse, poor parental bonding and attachment, and trauma (including bullying and post-traumatic disorder) compared with controls [35–40]. A recent systematic review of cross-sectional, prospective, and retrospective studies (*n* = 25) has documented a strong association between early childhood trauma and later schizotypy and suspiciousness (odds ratio range = 2.01 to 4.15), adjusting for basic demographics [41]. There is some evidence for emotional abuse, neglect, and stressful childhood events including bullying being especially strong predictors of schizotypy; however, on the whole, differential effects of trauma were not found. The ALSPAC prospective cohort study (*n* = 6437) found that 8- and 10-year-olds identified as victims of bullying reported significantly more psychotic symptoms at age 12 years (odds ratio = 1.94) and with even stronger associations when victimization experience was chronic and severe (odds ratio = 4.6). This finding was independent of prior psychopathology, cognitive functioning, and family adversity.

Prospective studies of attachment converge on the finding that poor parental bonding determined by a child showing great emotional distress (anxious attachment) or little distress avoidant attachment) in the absence of an attachment figure has been differentially linked with positive schizotypy and both positive and negative schizotypy, respectively [42]. In a systematic review of 22 studies of attachment and schizophrenia, Gumley et al. [43] found small to moderate effects between attachment insecurity and an increase in positive and negative symptoms of schizophrenia. However, there is a general consensus that study methodologies are heterogeneous with small samples and more longitudinal studies tracking the development beyond the early years of development are needed to establish long-term effects into adulthood.

In the maltreatment literature, neglect appears to be central in the development of schizotypy. In particular, victims of physical neglect have a 4.9-fold increase in SPD [44]. In one of the few prospective longitudinal child health studies in Mauritius, children being left “home alone” at age 3 years (*n* = 34) were compared to children cared for by siblings/relatives (*n* = 222) and children cared for by their mothers (*n* = 1498). Children left home alone scored significantly higher levels of psychotic symptoms and conduct disorder at age 17 years and schizotypy and crime at age 23 years accounting for social adversity and ethnicity (Wong KK, Raine A, & Venables P (under review). Being left home alone at age three years is associated with increased schizotypy and antisocial behavior at ages 17 and 23 years.)Why do psychosocial factors affect the development of schizotypal symptoms? There is some speculation that early abuse, neglect, and stress may result in structural and functional brain differences that give rise to schizotypal symptoms [45].

### Neurocognitive Functions and Brain Anatomy

Recent studies have examined neurocognitive impairments in schizotypy and suspiciousness. A review of these studies reveals that schizotypal individuals have neurocognitive impairments in executive functions, sustained attention, working memory, verbal and spatial learning and memory, latent inhibition, negative priming, hemisphere asymmetry, verbal fluency, and motor skills [6•, 9].

In paranoia, arguably the most well replicated finding is the stable characteristic of fast thinking (“jumping-to-conclusions”) and belief inflexibility where patients high in delusions make decisions based on limited information [46–48]. This abnormal reasoning bias is assessed using an established probabilistic reasoning task, the Beads Task [49], which has repeatedly distinguished patients with persecutory delusions from normal controls. However, the link between jumping-to-conclusions and suspicious young adults [50] and younger populations in the general population is weaker and less consistent [51, 52]. A comprehensive review of functional neuroimaging and neural network studies [53••], though predominantly cross-sectional in design, supports the jumping-to-conclusions cognitive deficit in patients with delusions and a presence of a reality distortion cluster of psychotic symptoms associated with various brain abnormalities (cerebral blood flow in the left lateral prefrontal cortex, ventral striatum, superior temporal gyrus, and parahippocampal region). There remains a gap in the literature as there is no longitudinal imaging study to date examining attributional bias or jumping-to-conclusion biases. A recent structural and functional brain imaging study of patients with persecutory delusions matched with controls found that patients showed brain abnormalities (reduced medial frontal/anterior cingulate cortex) in regions associated with the pathogenesis of delusions [54]. However, the study sample size was small (*n* = 22), patients with different subtypes of delusions were grouped together, and thus findings deserve to be replicated.

Brain-imaging studies of SPD report increased prefrontal activation as measured by EEG and the performance of schizotypals tends to fall between the range of normal controls and schizophrenia patients [55], though more recent studies suggest that schizotypals are more similar to controls but outperforms the schizophrenic patient group [56]. However, a few studies using functional magnetic resonance imaging (fMRI) and near-infrared optical spectroscopy (NIRS) methods have also documented enhanced creativity and divergent thinking in individuals high in schizotypy compared with low schizotypy [57] and in schizotypals compared to schizophrenics and healthy controls [58], which has been attributed to increased right hemisphere functioning and right prefrontal activation. Schizotypals perform poorly on facial emotional recognition tasks, struggle to label positive emotions, fail to think in another person’s perspective as assessed using theory of mind tasks, and over-respond to hostile/threatening stimuli [59, 60]. In terms of brain structure, a review of 17 studies found that SPD patients have abnormalities paralleling those found in schizophrenia patients in the superior temporal gyrus, parahippocampus, corpus callosum, thalamus, and septum pellucidum, as well as in total cerebrospinal fluid volume [61]. However, unlike schizophrenic patients, SPD patients showed normal functioning in the medial temporal lobes. On balance, the imaging findings suggest that SPD represents a milder form of disease along the schizophrenia continuum.

### Molecular Genetics

The identification of endophenotypes, the separation of behavioral symptoms into identifiable phenotypes with clear genetic connections, is a commonly used method to examine the underlying genetic causes of schizophrenia. A recent study investigated four candidate genes (*DTNBP1*, *NRG1*, *DAOA/G32*, and *DAAO*) common to schizophrenia and schizotypy and found DTNBP1, SNPs, rs2619522, and rs760761 single nucleotide polymorphisms (SNPs) to be associated with a decrease in positive and paranoid schizotypy scores [62•]. In particular, *DAAO* haplotype variability was noted on negative and disorganization schizotypy while DTNBP1 haplotype was associated with paranoid schizotypy.

The genetic studies on paranoia are more sparse. Only recently has the heritability estimates of paranoia been estimated in a large sample of twin pairs (*N* = 5059) from England and Wales, finding that 50% of the variability in paranoia in the general population is due to genes [63••]. Another recent finding from a Chinese sample showed moderate heritability in 2000 younger twin pairs (25 to 39%) [64•]. While some progress has been made to investigate genetic influences on paranoia, identifying the exact gene has proved more challenging and has yet to be conducted.

### Nutrition

Poor prenatal nutrition has been associated with schizophrenia spectrum disorders, specifically with schizotypal traits, in the Netherlands and China. In a sample of Mauritian children, Venables and Raine [65] established that poor nutrition at age 3 years (defined as anemia and stunting) was related to schizotypal traits at age 23 years—a relationship that was mediated by cognitive functioning (e.g., Verbal IQ and performance IQ) at age 11 years.

### Childhood Correlates of Schizotypy and Suspiciousness

Only a handful of studies to date have assessed schizotypy and suspiciousness in children and adolescents, and so childhood correlates are an underexplored area of research. In a large survey of the UK and Hong Kong schoolchildren (aged 8 to 14 years), children with high levels of suspiciousness reported significantly higher levels of anxiety, aggression, callous-unemotional traits, and lower levels of self-esteem compared with their non-suspicious peers, while self-reported suspicions were corroborated by peer ratings but not teacher or parent ratings, whether children’s suspicions are genuine or unfounded have yet to be tested [5••]. A short follow-up study on the same group of the suspicious and non-suspicious children found that self-reported persistent bullying experiences and hostile attributional biases predicted group membership (Wong, K. K. et al, unpublished data). A prospective study of paranoia in young children and adolescents: the role of hostile attributional bias and peer victimization.). Thematic analysis of the same sample confirmed that children with high levels of suspiciousness further identified children’s worries about their peers, which were corroborated by peer-rated suspiciousness (Wong KK (submitted). A qualitative study of childhood suspiciousness.).

## Treatment and Interventions

As the causes of schizotypal personality disorder are unclear, its treatment has also been understudied. One line of research extending the literature on brain deficiencies using carefully designed stratified randomized controlled trial showed that early environmental enrichment consisting of physical exercise, cognitive stimulation, and nutritional enhancement at 3–5 years both improved brain functioning at 11 years and reduced schizotypy at ages 17 and 23 years [66•]. This suggests that omega-3 as a nutritional supplementation could be helpful in reducing schizotypal traits in the long-run.

Recent published and ongoing randomized controlled (RCT) experiments on persecutory delusions targeting the cognitive causes of persecutory delusions have been promising. One of the first RCTs on persecutory delusions showed that six sessions of worry reduction cognitive behavioral therapy (CBT) intervention plus standard care completed over 8 weeks was better than standard care alone at reducing baseline worries and persecutory delusions at 8 weeks and follow-up at 24 weeks [67]. In another RCT, 30 patients were randomized into virtual CBT versus treatment as usual (TAU) and found that virtual CBT was as successful in reducing paranoid delusions overtime. While this study has a small sample and researchers were not blinded to patients’ treatment group, other ongoing RCT randomizing 360 patients into TAU and *SlowMo* intervention (a digital therapy targeting inflexible thinking in paranoia) or TAU alone is currently being investigated [68••].

## Summary and Future Directions

Our understanding of the etiology of schizotypy and suspiciousness has made significant progress over the years. Still, it is clear that more mediation analyses of prospective longitudinal studies and brain imaging studies (especially in delusions research), coupled with carefully designed randomized controlled trials, are needed to elucidate the etiology of schizotypy, suspiciousness, and ultimately schizophrenia. Though there have been some promising results from intervention studies, the evidence thus far stems largely from adult patients and so extending this line of work developmentally to younger populations may be effective in informing the development of early preventative interventions. More RCTs with longer follow-up may also be helpful in assessing the duration of intervention effects beyond the average follow-up period of an RCT. Finally, virtual reality as a non-invasive but immersive tool to assess and intervene on patients’ paranoid thoughts and other mental disorders holds great potential.
